# Nonlinear Spectroscopy in Chlorophyll Dimers Embedded in an Asymmetric Phonon Bath

**DOI:** 10.1021/acsphyschemau.4c00085

**Published:** 2025-05-24

**Authors:** Mohamad Toutounji

**Affiliations:** College of Science, Department of Chemistry, 11239 UAE University, P.O. Box 15551 Al-Ain, UAE

**Keywords:** photosynthetic complexes, 1-phonon profile asymmetry, linear and nonlinear spectra, excitonic coupling, exciton−phonon coupling, asymmetric spectral density, dephasing

## Abstract

The electronic transition dipole moment 4-point time correlation function for a dimeric photosynthetic complex, from which nonlinear optical time-domain signals may be obtained. This 4-point time correlation function draws on an experimentally fit spectral density of the surrounding phonons of the photosynthetic protein. The spectral density of the photosynthetic phonons renders a phonon-sideband characterized by its *asymmetry*, caused by the unequal contribution from the photosynthetic phonons (bath) to the low- and high-energy sides of the optical signals. This spectral density manifests its asymmetry explicitly in the 1-phonon profile, due to the intimate spectral connection between them, which will in turn reflect in the entire phononic part of the absorption spectrum. The asymmetry plays an important role in characterizing the exciton–phonon coupling strength and the phonon relaxation mechanism, thereby providing flexibility in modeling the degree of symmetry needed for the bath and imparting the capability of fine-tuning the nature of electron–phonon coupling caused by pigment–protein interaction. To this end, the obtained nonlinear optical electronic transition dipole moment time correlation functions (Liouville space pathways) whereby both excitonic and exciton–phonon couplings are accounted for are deemed convenient, more tractable, and computationally expedient, a unique advantageous feature in the case of a multimode system, which is often the case in photosynthetic complexes. Linear spectra and photon echo signals to probe pigment–protein complexes, in which pure electronic dephasing, vibrational relaxation effects, 1-phonon profile asymmetry, exciton–exciton coupling, and exciton–phonon coupling in bacterial reaction centers and photosynthetic complexes are provided.

## Graphical abstract

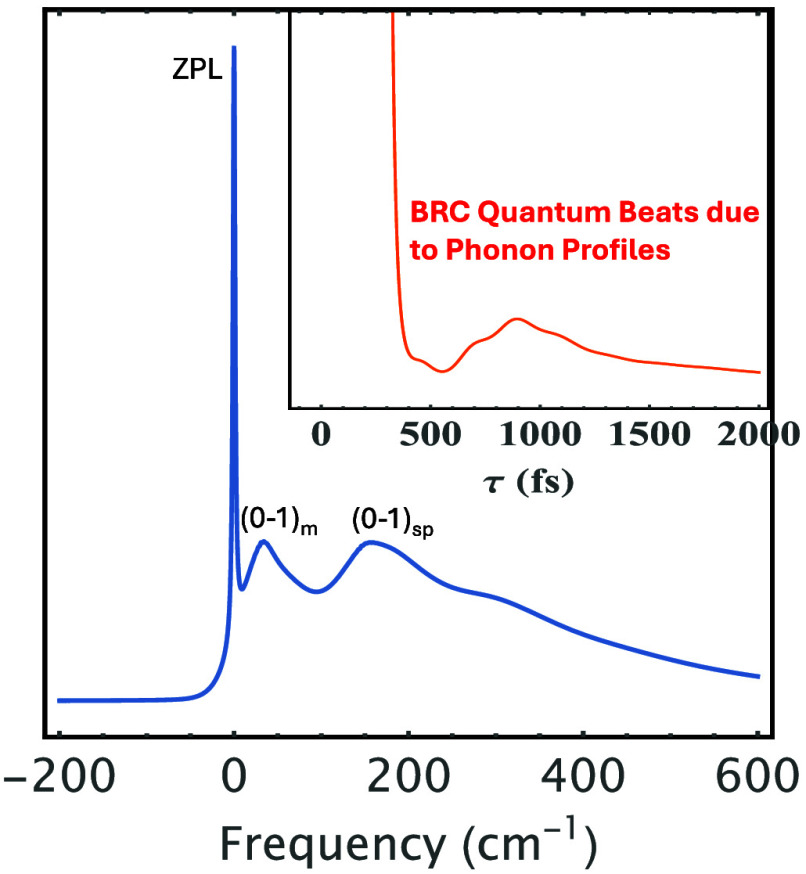

## I Introduction

Nonlinear optical spectra provide structural and dynamical information about the important properties of these systems in condensed media. These properties include exciton–exciton and electron–phonon couplings, electronic dephasing, phonon relaxation, echo peak shift (for extracting spectral density), and any other dynamical information. Although it is often difficult to obtain structural and dynamical information from linear spectroscopy in condensed systems, important peaks such as the zero-phonon line (ZPL) and phonon sideband (PSB) may be masked by static inhomogeneous broadening/dephasing. For this reason, more robust techniques, such as 4-wave mixing experiments, are required.^1–10^ The shape and width of spectral profiles of molecular aggregates, especially photosynthetic complexes, are caused by system-bath (pigment–protein) interaction, giving rise to dephasing and other dissipative forces.^1,2,7–16^

The employment of a physically accurate bath spectral density leads to correct accounting for these spectral and dynamical properties. While theoretical formulation under the Markovian and Condon approximations leads to symmetric phonon profiles, the experiments performed on molecular chromophores embedded in proteins, polymers, and glasses have shown *asymmetric* phonon profiles, most of which are dominated by 1-phonon profile.^6–9,14–18^ Many theoretical works utilize Debye spectral density or structurally similar spectral densities (e.g., exponential, Gaussian, the Brownian oscillator model, or Lorentzian function spectral densities, etc., all of which signify symmetric phonon bath distribution). These spectral densities are inherently symmetric and, therefore, do not seem to accurately model the spectra in bacterial reaction centers (RCs) and antenna complexes, leading to inaccurate shape and symmetry of the PSB in the line shape.^7–9,14–18^

Small et al.^6–8,19^ used experimental fit of hole-burned spectra at various temperatures on chlorophylls embedded in the and *Rhodopseudomonas* (*Rps*. *viridis*) RC protein complex to determine the strength of the linear electron–phonon coupling, defined by the Huang–Rhys factor (S), of the surrounding protein phonons in photosynthetic complexes and the associated 1-phonon profile. Hayes–Small formalism (HSF)^6–8,19^ had proposed, guided by their experimental data, that the protein phonons could be composed of a half Lorentzian function on the high-energy side and a half Gaussian function on the low-energy side in the frequency domain, as one possible way to account for the asymmetry in the phonons spectral density; hence the phonon bath distribution and 1-phonon profile. Henceforth, this asymmetric spectral density of phonons and the 1-phonon profile will be referred to as a G-L distribution. The G-L distribution will be adopted here in the time domain to account for the asymmetry in calculating nonlinear 4-wave mixing signals, e.g., photon echo. Small et al.^6–8,19^ and other research groups^9,14–18^ did experiments on chlorophylls embedded in the protein complex. They showed that their spectra may satisfactorily fit a G-L distribution in the frequency domain. Feng et al.^16^ reported calculations using parameters typically found in photosynthetic complexes and showed that using the G-L distribution to fit their spectral data was as gratifying as the log-normal distribution.

Recently, Toutounji^20,21^ reported a linear electronic transition dipole moment time correlation function based on pure electronic dephasing (Γ_ZPL_), linear electron–phonon coupling, and asymmetric PSB using a G-L distribution function. This correlation function is computationally expedient, mathematically tractable, and easily applicable to multimode systems.^22^ Finally, it resolves the discrepancies, errors, and inconsistencies arising when using HSF^6^ in multimode systems. The Fourier transform of the linear electronic transition dipole moment time correlation function derived by Toutounji^21^ yields an absorption line shape of one mode that is identical to that of HSF and that of Jankowiak et al.^17^ Calculations of spectra of *Rps*. *viridis* RC protein complex special pair (the mean frequency mode and the marker mode) were presented, showing excellent agreement. It is noteworthy that while the line shape function of Toutounji, that of HSF,^19^ and that of Jankowiak et al.^17^ are identical for *one vibrational mode*, and discrepancy arises only in case of more than one-mode system where HSF falls short and that of Toutounji^20,21^ and Jankowiak et al.^17^ yield identical line shape functions.^21^ Although Jankowiak et al.^17^ corrected the discrepancies and inconsistencies caused by HSF in the case of a multimode system, they made the line shape expression more complicated and less efficient computationally. Additionally, the expansion terms of the nested sums in their line shape expression keep growing as the pigment-complex coupling strength (S) increases, which requires more phonon progression terms, especially at high temperatures, all of which are avoided using the formalism of Toutounji.^20,21^ Furthermore, Jankowiak et al.^17^ did not seem to have reported any numerical strategy for terminating these proportionally growing expansion terms, causing accuracy issues to arise.

The present work reports a linear electronic transition dipole moment time correlation function whereby Γ_ZPL_, exciton–exciton coupling, linear exciton–phonon coupling, and *asymmetric* PSB using a G-L distribution function are all accounted for in pigment–protein complexes. The exciton–exciton coupling manifests itself in its most basic form through blue shifting the vibronic bands: only the upper excitonic state energy shows up in the spectra in accord with the formula, *E*_+_ = ε + *V*, where ε and *V* the Franck–Condon electronic energy transition and resonant pigment–pigment coupling (excitonic coupling), respectively. This excitonic coupling *V* is assumed to be larger than the electron–phonon coupling (through the reorganizational energy λ) in the current formulation to reflect some *exciton formation* to the optical signal. Note that neither disorder nor exchange coupling effects are accounted for. The dimerization shifting effect (often called energy displacement) will also be precluded in this formulation.

Additionally, this work shows how to compute nonlinear optical signals (4-wave mixing experiments) of systems embedded in an asymmetric bath of phonons of the nonlinear optical electronic transition dipole moment correlation functions (Liouville space pathways) in the time domain to probe pigment–protein complexes, in which the pure electronic dephasing (Γ_ZPL_), vibrational relaxation effects, 1-phonon profile asymmetry, exciton–exciton coupling, and exciton–phonon coupling in bacterial RCs are accurate. Although Jankowiak et al.^17^ corrected the discrepancies and deficiencies posed by HSF, not only is the pigment–pigment interaction (*V*) missing, but also extending it to the time domain is infeasible due to its complexity (computationally demanding, particularly in an N-mode system), especially in case of a strong exciton–phonon coupling where S is large, which requires many terms, vide supra, rendering computational inefficiency. The nonlinear electronic transition dipole moment 4-point time correlation function for a dimeric photosynthetic complex whereby the surrounding phonons exhibit *asymmetric* distributions at different temperatures is derived, starting from the time domain. Although formulas for optical signals, e.g., photon echo, pump–probe, and hole-burning signals, are provided, this work only reports computations for photon-echo optical signals. Note that neither HSF nor that of Jankowiak and Reppert^17^ would readily lead to any time-domain 4-wave mixing signals, especially for multimode systems. Additionally, the lineshaped expression of Jankowiak and Reppert^17^ does not account for exciton–exciton coupling.

Electron–phonon coupling competes appreciably in molecular aggregates with electronic delocalization (caused by excitonic coupling). While excitonic coupling tends to drive electronic delocalization, the interaction between electronic degrees of freedom and vibrations seems to support the converse, that is, suppressing it by localizing it. This interplay between the two couplings tends to affect excitonic transport through the aggregates. The spectral density distribution of the vibrations (phonons) plays a central role in this competition. Major parts of an optical signal depend on the form and shape of the spectral density. For example, the phonons reorganizational energy, λ, is expressed in terms of the spectral density, *C*(ω), as λ = *∫*_0_^∞^ *C*(ω)/*ω*dω while λ and *C*(ω) quantify electron–phonon coupling strength, *V* signifies the Coulombic pigment–pigment electronic coupling in photosynthetic pigment–protein complexes (PPCs). *V* and λ are two fundamental factors that play a crucial role in driving excitation transfer. While *V* signifies the pigment–pigment coupling, λ (the bath reorganization energy) measures pigment-phonon coupling strength through the above integral formula, vide supra. For this reason, many groups have been studying the interplay between these two them, thereby understanding quantum coherence longevity and its impact on electronic energy transfer. The case whereby *V* < λ leads to Förster’s theory, whereas the opposite limit (*V* > λ) can be treated using Redfield theory. (The former case results in fast dephasing or incoherent transfer caused by electron–phonon coupling in which exciton wave function would be incomplete). This case results in fast dephasing, which hinders exciton formation. However, the intermediate regime where *V* and λ are comparable is more challenging and requires different treatments. Different approaches have been developed to treat the above regimes using Redfield or modified Redfield equations and their polaron-transformed versions, including relaxing the secular and Markovian approximations.

This article is organized as follows. Section 2 provides a brief background of the linear spectroscopic regime in the time domain to probe pigment–protein complexes, in which the pure electronic dephasing, vibrational relaxation effects, 1-phonon profile asymmetry, exciton–exciton coupling, and exciton–phonon coupling in bacterial RCs and photosynthetic PPCs. Nonlinear electronic transition dipole moment-time correlation functions are derived in Section 3, whereby 4-wave mixing signals may be computed. Section 4 provides calculations of linear homogeneous spectra and photon echo signals. Section 5 provides concluding remarks.

## II Theoretical Background for Linear Response in Dimeric Complexes

This Section will briefly review our derivation^20^ of linear electronic transition dipole moment time correlation function of homodimers. Starting with introducing some terms for a single monomer m, one can define *B*_*m*_ = |*gm*⟩⟨*em*| and *B*_*m*_^†^ = |*em*⟩⟨*gm*|, resulting in *B*_*m*_^†^ *B*_*m*_ = |*em*⟩⟨*em*| and *B*_*m*_ *B*_*m*_^†^ = |*gm*⟩⟨*gm*|. This leads to expressing the diagonal adiabatic electronic Hamiltonian for a monomer *m*

1 $$H_{\mathrm{el}}\left(m\right)=H_{\mathrm{gm}}{B_{m}B}_{m}^{†}+H_{\mathrm{em}}B_{m}^{†}B_{m}$$

with *H*_*gm*_ and *H*_*em*_ being the ground and excited states nuclear Hamiltonians. For example, while the ground-state nuclear Hamiltonian of *H*_*gm*_ may assume the form

1a $$H_{\mathrm{gm}}=\hbar \omega_{\mathrm{jm}}b_{\mathrm{jm}}^{†}b_{\mathrm{jm}}$$

the excited-state nuclear Hamiltonian *H*_*em*_ of monomer m

1b $$H_{\mathrm{em}}=\hbar \omega_{\mathrm{jm}}b_{\mathrm{jm}}^{†}b_{\mathrm{jm}}+\hbar \omega_{\mathrm{jm}}\left(b_{\mathrm{jm}}^{†}+b_{\mathrm{jm}}\right)d_{\mathrm{jm}}/\sqrt{2}+\hbar \omega_{\mathrm{jm}}{d_{\mathrm{jm}}}^{2}/2$$

Here, the upper linear displacement *d*_*jm*_, ω_*jm*_ is the frequency, and *b*_*jm*_^†^(*b*_*jm*_) the creation (annihilation) boson operators of the vibrational mode *j* of monomer *m* due to linear electron–phonon coupling. Consider an excitonically coupled dimer with nuclear vibrations coupled to a bath of phonons. Assuming a homodimer in which the monomers (pigments) are coupled through the dipole–dipole effect, the Hamiltonian of the homodimer may assume the form

2 $$H=\sum_{m=1}^{2}\varepsilon_{m}B_{m}^{†}B_{m}+\sum_{\mathrm{mn}}V_{\mathrm{mn}}B_{m}^{†}B_{n}+H_{\mathrm{bath}}+H_{\mathrm{pigment}‐\mathrm{bath}}$$

with

2a $$H_{\mathrm{pigment}‐\mathrm{bath}}=\sum_{j}\sum_{m=1}^{2}\eta_{\mathrm{mj}}B_{m}^{†}B_{m}Q_{j}$$

where ε_*m*_ is the electronic transition energy of pigment m, *B*_*m*_^†^ and *B*_*m*_ are the electronic excitation and deexcitation Fermi operators, respectively, η_*jm*_ is pigment-bath coupling constant, *V*_*mn*_ is the Coulombic coupling between the electronic charge densities of the monomers n and m that make up the dimers (no Coulombic exchange interaction is considered here), and *Q*_*m*_ is the collective bath coordinate coupled to monomer *m*. The form of the bath will not be important here, and the dynamics will be related to the spectral density *C*(ω) associated with each monomer m. In other words, the bath effect will be considered through the G-L distribution of the spectral density, which will be employed later.

The derivation of a linear electronic transition dipole moment time correlation function in which the exciton–phonon coupling is accounted for will be briefly sketched herein, rendering overtones that fold themselves on the fundamental transition (1-phonon profile) that has an *asymmetric* shape made of half Gaussian on the red side and half Lorentzian on the high-energy side, as reported by Hayes-Small and others.^9,14–18^ Starting with the definition of the linear electronic transition dipole moment time correlation function *J*(*t*)­

3 J(t)=⟨μ(t)μ(0)⟩

where the electronic dipole moment is μ­(*t*) = ∑_*m*_μ_*m*_(*B*_*m*_ + *B*_*m*_^†^) in which μ_*m*_ is the dipole moment operator of the monomer m. The linear electronic transition dipole moment time correlation function in the Heisenberg representation is given by

4 $$J\left(t\right)=\mathrm{Tr}\left[e^{\mathrm{iHt}/\hbar }\mu \left(0\right)e^{-\mathrm{iHt}/\hbar }\mu \left(0\right)\rho_{\mathrm{eq}}\right]$$

in which the equilibrium density matrix is ρ_eq_ given by

5 $$\rho_{\mathrm{eq}}=\frac{e^{-\beta H}|g\rangle \langle g|}{Z}$$

where *Z* is the canonical partition function. The trace in eq 7 is taken over electronic, nuclear, and bath degrees of freedom. Expanding eq 7 in the electronic basis will project *J*(*t*) over in the nuclear and bath subspaces, whereas tracing over vibrations will give rise to FCFs, of a linearly coupled system, and tracing over the bath degrees of freedom will lead to the spectral profile shape and broadening.

Different research groups^14–18^ have fitted their data using a 1-phonon profile of an asymmetric shape of their photosynthetic complexes; the 1-phonon profile, whose width builds on the ZPL width (electronic dephasing), acts as the building block of the subsequent transitions, making up the PSB in the system of interest. This asymmetric 1-phonon profile may plausibly adopt this form

6 $$I_{1}\left(\omega \right)=\left[u\left(\omega -\omega_{j}\right)\frac{\gamma_{j}/2\pi }{{\left(\omega -\omega_{j}\right)}^{2}+{\left(\gamma_{j}/2\right)}^{2}}+\frac{1}{\sqrt{2\pi \sigma_{j}^{2}}}u\left(\omega_{j}-\omega \right)e^{-{\left(\omega -\omega_{j}\right)}^{2}/2\sigma_{j}^{2}}\right]$$

rendering a spectral density *C*(ω)= *S*_*j*_ *I*_1_(ω), with *S*_*j*_ being Huang–Rhys factor, where γ_*j*_ denotes the full width at half-maximum (fwhm) of the Lorentzian profile associated with mode *j*, whereas σ_*j*_ is the corresponding standard deviation of the Gaussian distribution, yielding $\mathrm{fwhm}=2\sigma_{j}\sqrt{2\ln2}$ . While the unit step function *u*(ω – ω_*j*_) ensures half of Lorentzian does not act earlier than ω_*j*_, $u\left(\omega_{j}-\omega \right)e^{-{\left(\omega -\omega_{j}\right)}^{2}/2\sigma_{j}^{2}}$ takes over before the mode frequency ω_*j*_, both of which carry different widths while composing one profile, resulting in an asymmetric shape. This asymmetry is also carried over to the spectral density *C*(ω). The pigments in the homodimer are assumed to have the same local *C*(ω), whereby fluctuations of both sites energy are uncorrelated, i.e., the spectral density is site independent. In short, *C*_*mn*_(ω) = δ_*mn*_ *C*_*m*_(ω) = *C*(ω). Performing the inverse Fourier transform of the 1-phonon profile of mode *j* yields the dephasing function *R*_*j*_(*t*) of the protein phonons for as^21^

7 $${R_{j}\left(t\right)=F}^{-1}\left[I_{1}\left(\omega \right)\right]$$

where

7a $$R_{j}\left(t\right)=L_{j}\left(t\right)+G_{j}\left(t\right)$$

with

7b $$L_{j}\left(t\right)=\frac{1}{2\pi }e^{-i\omega_{j}t}\left\{ie^{\gamma_{j}t/2}\mathrm{Ei}\left(-\frac{\gamma_{j}}{2}t\right)+e^{-\gamma_{j}t/2}\left[\pi -i\mathrm{Ei}\left(\frac{\gamma_{j}}{2}t\right)\right]\right\}$$

7c $$G_{j}\left(t\right)=\frac{1}{2}e^{-i\omega_{j}t}e^{-\sigma_{j}^{2}t^{2}/2}\left(1+E\mathrm{rf}\left(\frac{i\sigma_{j}t}{\sqrt{2}}\right)\right)$$

and Erf (.) in eq 13 is the error function. Note 1/γ_*j*_ is the phonon relaxation time and $\sigma_{j}=\gamma_{j}/2\sqrt{2\ln2}$ such that the 1-phonon profile width (fwhm) is given by Γ_*j*_ = (γ_*j*_ + σ_*j*_)/2. Both γ_*j*_ and σ_*j*_ values, and hence Γ_*j*_, may be chosen to fit experimental data, as reported in ref [ ^20,21^ ]. Figure 1 shows the spectral density function using G-L distribution with various values of γ_*j*_ and σ_*j*_ while holding ω_*j*_ and *S*_*j*_ constant to exhibit the asymmetry. All panels a–d in Figure 1 were calculated at ω_*j*_ = 0 and *S*_*j*_ = 1. Figure 1 shows how the asymmetry variably changes as one goes from panel a–d, where panel a is the most asymmetric and panel d is reasonably symmetric. This degree of freedom in choosing symmetry/asymmetry is valuable in simulating open systems. Thus, this form of G-L distribution has the capability of fine-tuning the nature of electron–phonon coupling.

**Figure 1:**
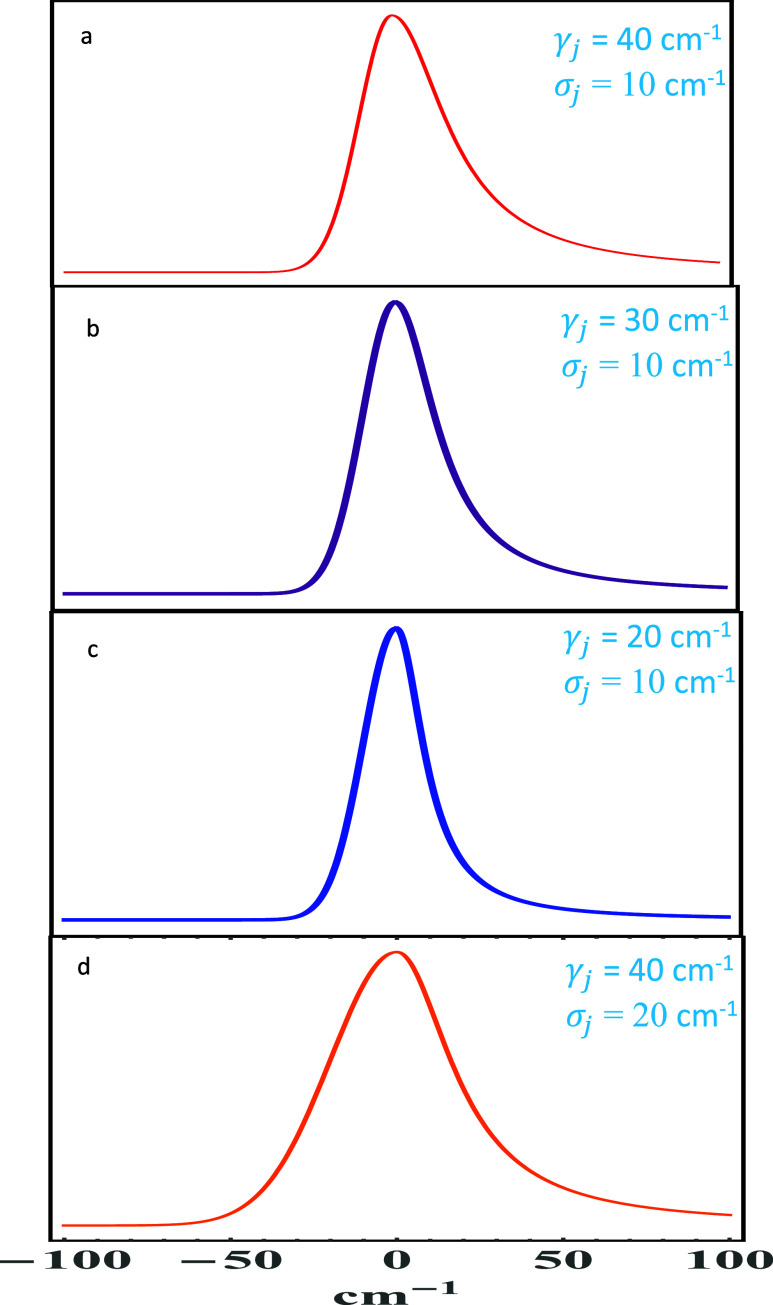
Spectral density function with different asymmetric shapes calculated using G-L distribution with the shown values of γ*_j_* and σ_*j*_ while holding ω_*j*_ = 0 cm^–1^ and *S*_*j*_ = 1.0, rendering a width (fwhm) of the 1-phonon profile transition given by Γ_*j*_ = (γ_*j*_ + σ_*j*_)/2. While panel (a) shows the most asymmetric spectral density and panel (d) represent a symmetric one, panels (b) and (c) are intermediate cases, thereby providing flexibility in modeling the degree of symmetry needed for the bath, and imparting capability of fine-tuning the nature of electron–phonon coupling caused by pigment–protein interaction.

The linear electronic transition dipole moment time correlation function that treats the electronic dephasing and phonon relaxation processes independently in the monomers *n* and *m* of mode *j* is given by^20^

8 $$J_{j}\left(t\right)=\exp\left[-i\varepsilon_{\mathrm{nm}}t\delta_{\mathrm{nm}}-\mathrm{iVt}-S_{j}\left(1-R_{j}\left(t\right)\right)-\Gamma_{\mathrm{ZPL}}\left|t\right|/2\right]$$

where 1/Γ_ZPL_ is the electronic pure dephasing time. The linear electronic transition dipole moment time correlation function in eq 14 distinguishes between electronic dephasing and vibrational relaxation times. As such, it is sensitive to both electronic dephasing and vibrational relaxation processes, where the vibrational relaxation process encompasses unequal relaxation times, 1/γ_*j*_ and 1/σ_*j*_, where the former is exponential decay and the latter Gaussian, leading to asymmetric PSB in pigment–protein complexes.^20,21^ The reader is referred to refs [ ^20,21^ ] for the dephasing function *R*_*j*_(*t*) role, utility, and significance and how the 1-phonon profile is obtained in the frequency domain, which is essentially the spectral density of bath phonons. It is the spectral density, which represents coupling strength and density of states of phonons, that imparts information about broadening and dephasing effects.

The linear electronic transition dipole moment time correlation function with temperature dependence using^3^

9 $$J\left(t\right)=\exp\left[-\int_{-\infty }^{\infty }\frac{1}{2\pi \omega^{2}}\left(\coth\left(\frac{\beta \hbar \omega }{2}\right)\left(1-\cos\left(\omega t\right)\right)+i\sin\left(\omega t\right)\right)C\left(\omega \right)d\omega \right]$$

for mode *j* is

10 $$J_{j}\left(t\right)=\exp\left\{-i\varepsilon_{\mathrm{nm}}t\delta_{\mathrm{nm}}-\mathrm{iVt}-\frac{\Gamma_{\mathrm{ZPL}}\left|t\right|}{2}-\Xi_{j}\left(t\right)\right\}$$

with

10a $${\Xi_{j}\left(t\right)=S}_{j}\left\{\coth\left(\frac{\beta \hbar \omega_{j}}{2}\right)\left[1-e^{i\omega_{j}t}R_{j}\left(t\right)\cos\left(\omega_{j}t\right)\right]+ie^{i\omega_{j}t}R_{j}\left(t\right)\sin\left(\omega_{j}t\right)\right\}$$

The absorption line shape rendered by eq 16 will result in a Lorentzian ZPL profile of width Γ_ZPL_ (fwhm) and multiphonon transitions making up the PSB of mode j with half-Gaussian and half-Lorentzian distributions of widths equal to σ_*j*_√*k* on the low energy side and *k*γ_*j*_ on the high energy side, respectively, where k is the number of phonons associated with the transition. Finally, the linear electronic transition dipole moment time correlation function for a multimode system in which the PSB displays an asymmetry is

11 $$J\left(t\right)=\exp\left\{-\frac{\Gamma_{\mathrm{ZPL}}\left|t\right|}{2}-\sum_{j=1}^{N}\left[i\varepsilon_{\mathrm{nm}}t\delta_{\mathrm{nm}}+\mathrm{iVt}+\Xi_{j}\right]\right\}$$

eq 18 represents the final form of the linear electronic transition dipole moment time correlation function of excitonically coupled pigments at finite temperatures for a multimode system, assuming no population transfer. In light of the above, eq 18 features (i) a homogeneous absorption spectrum that accounts for the asymmetric shape of the PSB and the symmetry of the ZPL due to electron–phonon coupling, (ii) pigment–pigment coupling interaction (excitonic coupling) for an N-mode system, (iii) the 1-phonon profile that carries its width over the overtones through folding, (iv) the elimination of all the inconsistencies, deficiencies, and difficulties associated with the theory presented by HSF, and, finally, (v) it is easily extendable to nonlinear spectroscopy, as will be demonstrated in the next Section.

## III Nonlinear Spectroscopy in Dimeric Complexes

This Section will extend the above linear spectroscopy over to nonlinear optical signals using a nonlinear dipole moment time correlation function approach whereby three subsequent laser pulses will be employed at different time intervals to polarize the molecular sample of interest. Starting with the third-order response S^(3)^(*t*_1_,*t*_2_,*t*_3_)^4^

12 S(3)(t3,t2,t1)=2ℏ−3Im∑α=14Rα(t3,t2,t1)

*R*_α_ represents the nonlinear correlation function, each of which represents the Liouville space pathway, and *t*_1_, *t*_2_, and *t*_3_ are the interaction intervals between the molecular sample and the radiation field. Using the fact that the correlation function is invariant under time translation, the correlation functions {*R*_α_(*t*_1_,*t*_2_,*t*_3_)}_α=1_^4^ are given by^4^

13 $$\begin{array}{l} R_{1}\left(t_{3},t_{2},t_{1}\right)=F\left(t_{1},t_{1}+t_{2},t_{1}+t_{2}+t_{3},0\right)\\ R_{2}\left(t_{3},t_{2},t_{1}\right)=F\left(0,t_{1}+t_{2},t_{1}+t_{2}+t_{3},t_{1}\right)\\ R_{3}\left(t_{3},t_{2},t_{1}\right)=F\left(0,t_{1},t_{1}+t_{2}+t_{3},t_{1}+t_{2}\right)\\ R_{4}\left(t_{3},t_{2},t_{1}\right)=F\left(t_{1}+t_{2}+t_{3},t_{1}+t_{2},t_{1},0\right) \end{array}$$

where the 4-point electronic transition dipole moment time correlation function *F*(τ_1_,τ_2_,τ_3_,τ_4_) in the Heisenberg picture reads

14 $$F\left(\tau_{1},\tau_{2},\tau_{3},\tau_{4}\right)=\left\langle e^{\mathrm{iH}\tau_{1}/\hbar }P\left(0\right)e^{-\mathrm{iH}\tau_{1}/\hbar }e^{\mathrm{iH}\tau_{2}/\hbar }P\left(0\right)e^{-\mathrm{iH}\tau_{2}/\hbar }e^{\mathrm{iH}\tau_{3}/\hbar }P\left(0\right)e^{-\mathrm{iH}\tau_{3}/\hbar }e^{\mathrm{iH}\tau_{4}/\hbar }P\left(0\right)e^{-\mathrm{iH}\tau_{4}/\hbar }\right\rangle$$

Invoking the Condon approximation, eq 21 becomes

15 $$F\left(\tau_{1},\tau_{2},\tau_{3},\tau_{4}\right)=\left\langle e^{\mathrm{iH}\tau_{1}/\hbar }e^{-\mathrm{iH}\tau_{1}/\hbar }e^{\mathrm{iH}\tau_{2}/\hbar }e^{-\mathrm{iH}\tau_{2}/\hbar }e^{\mathrm{iH}\tau_{3}/\hbar }e^{-\mathrm{iH}\tau_{3}/\hbar }e^{\mathrm{iH}\tau_{4}/\hbar }e^{-\mathrm{iH}\tau_{4}/\hbar }\right\rangle =\mathrm{Tr}\left\{e^{\mathrm{iH}\tau_{1}/\hbar }e^{-\mathrm{iH}\tau_{1}/\hbar }e^{\mathrm{iH}\tau_{2}/\hbar }e^{-\mathrm{iH}\tau_{2}/\hbar }e^{\mathrm{iH}\tau_{3}/\hbar }e^{-\mathrm{iH}\tau_{3}/\hbar }e^{\mathrm{iH}\tau_{4}/\hbar }e^{-\mathrm{iH}\tau_{4}/\hbar }\rho_{g}\right\}$$

Evaluating the trace over excitonic, nuclear, and bath degrees of freedom yields

16 $$\begin{array}{l} F\left(\tau_{1},\tau_{2},\tau_{3},\tau_{4}\right)=\exp\left\{-\left[i\varepsilon_{\mathrm{mn}}\delta_{\mathrm{mn}}+\mathrm{iV}+\frac{\Gamma_{\mathrm{ZPL}}}{2}\right]s_{1}-\Xi_{j}\left(s_{1}\right)\right\}\\ \times \exp\left\{\left[i\varepsilon_{\mathrm{mn}}\delta_{\mathrm{mn}}+\mathrm{iV}+\frac{\Gamma_{\mathrm{ZPL}}}{2}\right]s_{2}+\Xi_{j}\left(s_{2}\right)\right\}\\ \times \exp\left\{-\left[i\varepsilon_{\mathrm{mn}}\delta_{\mathrm{mn}}+\mathrm{iV}+\frac{\Gamma_{\mathrm{ZPL}}}{2}\right]s_{3}-\Xi_{j}\left(s_{3}\right)\right\}\\ \times \exp\left\{-\left[i\varepsilon_{\mathrm{mn}}\delta_{\mathrm{mn}}+\mathrm{iV}+\frac{\Gamma_{\mathrm{ZPL}}}{2}\right]s_{4}-\Xi_{j}\left(s_{4}\right)\right\}\\ \times \exp\left\{\left[i\varepsilon_{\mathrm{mn}}\delta_{\mathrm{mn}}+\mathrm{iV}+\frac{\Gamma_{\mathrm{ZPL}}}{2}\right]s_{5}+\Xi_{j}\left(s_{5}\right)\right\}\\ \times \exp\left\{-\left[i\varepsilon_{\mathrm{mn}}\delta_{\mathrm{mn}}+\mathrm{iV}+\frac{\Gamma_{\mathrm{ZPL}}}{2}\right]s_{6}-\Xi_{j}\left(s_{6}\right)\right\} \end{array}$$

with

16a $$\begin{array}{l} s_{1}=\tau_{1}-\tau_{2}\\ s_{2}=\tau_{1}-\tau_{3}\\ s_{3}=\tau_{1}-\tau_{4}\\ s_{4}=\tau_{2}-\tau_{3}\\ {s_{5}=\tau }_{2}-\tau_{4}\\ s_{6}=\tau_{3}-\tau_{4} \end{array}$$

Now that an expression of *F*(τ_1_,τ_2_,τ_3_,τ_4_) is developed at various time intervals for a complex dimer with excitonic and electron–phonon couplings, one can readily find any of {*R*_α_(*t*_1_,*t*_2_,*t*_3_)}_α=1_^4^ with the aid of eq 20. This will allow the calculation of *all* nonlinear optical signals, of which examples are provided below.

For example, define the echo response function $R\left(t_{3},t_{2},t_{1}\right)$ as

17 $$R\left(t_{3},t_{2},t_{1}\right)\equiv R_{2}\left(t_{3},t_{2},t_{1}\right)+R_{3}\left(t_{3},t_{2},t_{1}\right)$$

whereby the optical stimulated photon echo signal (SPE), assuming impulsive (infinitely short) laser pulses, can be calculated using eqs 20–23

18 $$S_{\mathrm{SPE}}\left(\tau^{\prime },\tau \right)=\int_{0}^{\infty }dt{\left|R\left(t,\tau ,\tau^{\prime }\right)\right|}^{2}{\left|\chi \left(t-\tau^{\prime }\right)\right|}^{2}$$

where τ′ is the delay time between the first and second pulses and τ is the delay between the second and third pulses. The SPE signal is one of the tools for probing *echo peak shift*, which measures the strength of electron–phonon coupling. While $R\left(t_{3},t_{2},t_{1}\right)$ governs the homogeneous *dynamical* contribution to the dephasing, χ­(*t*_3_– *t*_1_) governs the static inhomogeneous *static* part. Another example is the integrated signal of the impulsive 2-pulse echo (IPE)

19 $$S_{\mathrm{IPE}}\left(\tau^{\prime }\right)=\int_{0}^{\infty }dt{\left|R\left(t,0,\tau^{\prime }\right)\right|}^{2}{\left|\chi \left(t-\tau^{\prime }\right)\right|}^{2}$$

whereas the time-resolved IPE signal is

20 $$S_{\mathrm{IPE}}\left(t,\tau^{\prime };T\right)={\left|R\left(t,0,\tau^{\prime }\right)\right|}^{2}{\left|\chi \left(t-\tau^{\prime }\right)\right|}^{2}$$

eqs 20–23 play a central role in finding nonlinear signals. Figure 2 utilizes the spectral density in eq 9 to calculate time-resolved 2-PE signal (top panel) using eq 28, integrated 2-PE signal (middle panel) using eq 27, and the corresponding homogeneous absorption spectrum (bottom panel) with the following parameters: Γ_ZPL_ = 3 cm^–1^, *T* = 2 K, γ*_j_* = 20 cm^–1^, σ_*j*_ = 4 cm^–1^, ω_*j*_ = 60 cm^–1^, and *S*_*j*_ = 0.50. All calculations reported herein will have an inhomogeneous broadening Δ ∼ 107cm^–1^. Figure 2 demonstrates the intimate relationship between the spectral density shape, width, and symmetry of those of both 1-phonon profile (0–1 transition) and fundamental quantum beat (labeled Beat1), thereby showing how asymmetry of the bath spectral density *C*(ω) manifests itself in PSB shape and phonon relaxation of the system at hand. Similarly, the 0–2 transition and Beat 2 are asymmetric as well. Figure 3 runs the same calculation using the same parameters as in Figure 2 but using a symmetric (Lorentzian) spectral density for contrast purposes, except the phonon Lorentzian damping was set γ_*j*_ = 10 cm^–1^ for more conspicuous results. The ZPL intensity has been chopped off for better clarity of the phonon profiles. Clearly, both the 0–1 transition and Beat1 appear *symmetric*, as do the 0–2 transition and Beat 2; this symmetry is ascribed to the employed symmetric spectral density.

**Figure 2:**
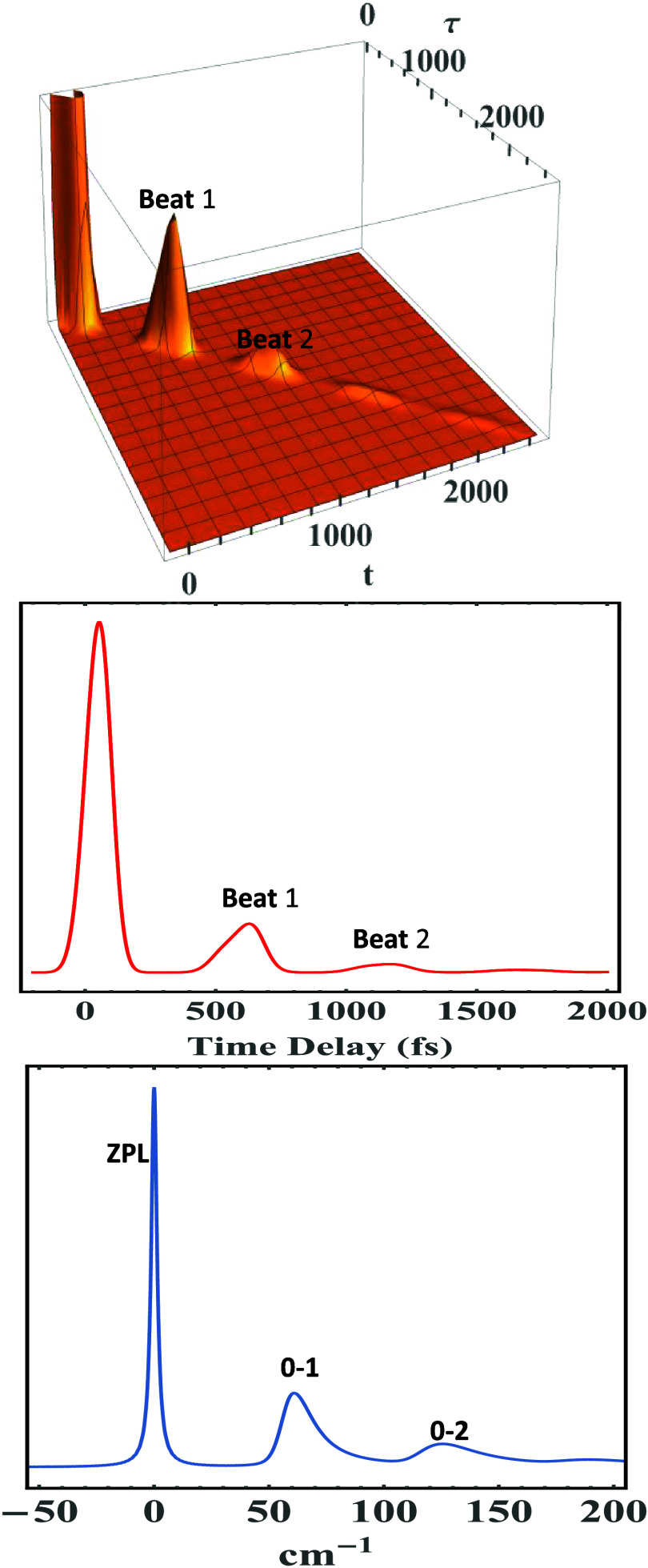
Time-resolved 2-pulse photon echo (PE) (top panel), its integrated signal (middle panel), and corresponding absorption spectrum (bottom panel) of a model system calculated with the parameters Γ_ZPL_ = 3 cm^–1^, *T* = 2 K, Δ ∼ 107 cm^–1^, γ_*j*_ = 20 cm^–1^, σ_*j*_ = 4 cm^–1^, ω_*j*_ = 60 cm^–1^, and *S*_*j*_ = 0.50 to show how PSB asymmetry manifests itself in quantum beats and phonon profiles.

**Figure 3:**
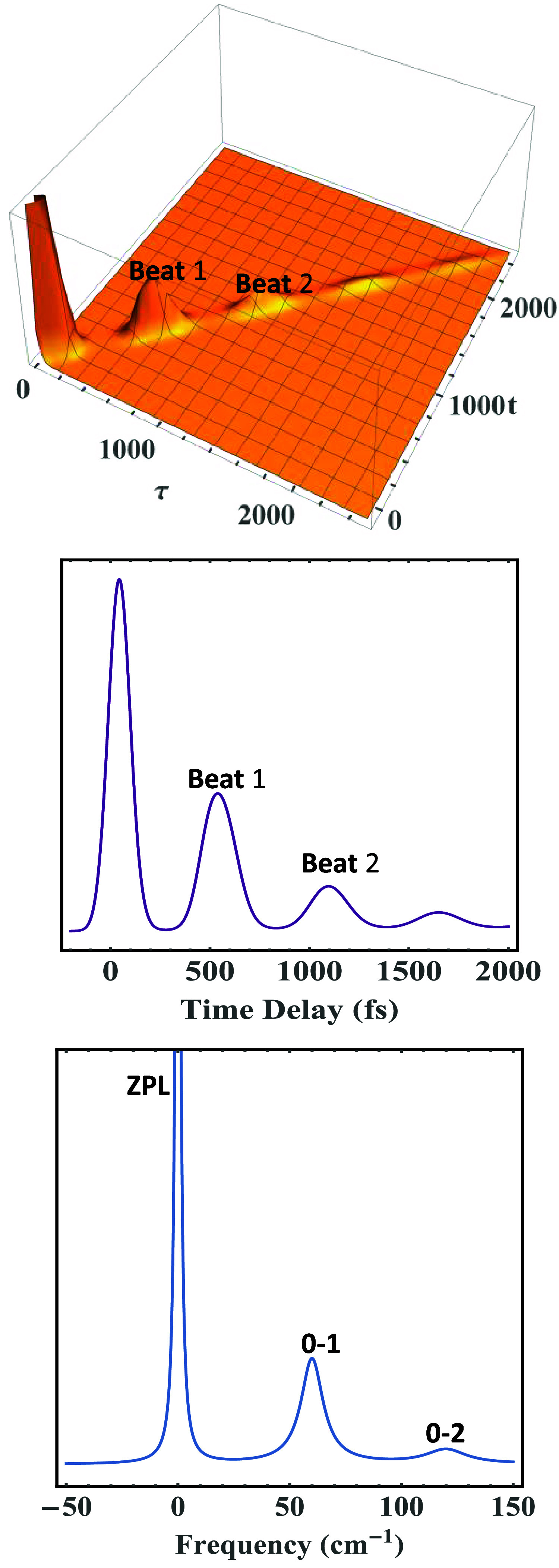
Time-resolved 2-pulse photon echo (PE) (top panel), its integrated signal (middle panel), and corresponding absorption spectrum (bottom panel) of a model system calculated with the parameters Γ_ZPL_ = 3 cm^–1^, *T* = 2 K, Δ ∼ 107 cm^–1^, γ_*j*_ = 10 cm^–1^, ω*_j_* = 60 cm^–1^, and *S*_*j*_ = 0.50 with Lorentzian (symmetric) spectral density, causing both 0–1 transition and Beat1 to appear perfectly *symmetric*, as do 0–2 transition and Beat 2. The ZPL intensity has been cut off for a better clarity of the phonon profiles, unlike those in Figure 2.

Hole-burning signal can also be calculated using eqs 20–23 as^4^

21 $$\begin{array}{l} S_{\mathrm{HB}}\left(\omega_{B},\omega_{L},\tau \right)=2{\left(\frac{1}{\hbar }\right)}^{3}\omega_{L}\int_{0}^{\infty }dt_{1}\int_{0}^{\infty }dt_{3}\{\chi \left(t_{3}+t_{1}\right)e^{i\omega_{L}t_{3}+i\omega_{B}t_{1}}[R_{1}\left(t_{3},\tau ,t_{1}\right)+\\ R_{4}\left(t_{3},\tau ,t_{1}\right)]+\chi \left(t_{3}-t_{1}\right)e^{i\omega_{L}t_{3}-i\omega_{B}t_{1}}\left(R_{2}\left(t_{3},\tau ,t_{1}\right)+R_{3}\left(t_{3},\tau ,t_{1}\right)\right)\} \end{array}$$

where ω_B_ is the burn frequency and ω_L_ is the light frequency. A final example of a spectral signal that may be calculated using eqs 20–23 is the impulsive pump probe signal^4^

22 SIPP(ωL,τ)=Re∫0∞dt3⁡eiωt3χ(t3)[R2(t3,τ,0)+R3(t3,τ,0]

Taking into account the above, one can see how HSF or that of Jankowiak et al.^17^ (both expressions represent eigenstate expansion) would not lend themselves to use in computing *any* of the nonlinear electronic transition dipole moment time correlation function except for hole-burning or fluorescence line narrowing spectra for a very limited number of modes, thereby reflecting the utility, feasibility, and efficiency of eqs 18 and 23 in calculating the above nonlinear signals. Although those of HSF and Jankowiak et al.^17^ do not offer the capability to compute any of these nonlinear signals in the time domain, eqs 19 and 23 may readily be utilized to perform these calculations using *nonlinear optical response theory*, vide supra.

## IV Calculations and Discussion

The local structural heterogeneity in the surrounding environment exhibits variation from one system to another. This structural heterogeneity hides the rich homogeneous structure of the chromophore. For this reason, one must resort to nonlinear optical experiments in order to reveal this hidden structure by unmasking it. While this heterogeneous structure manifests itself in frequency-domain experiments as static inhomogeneous spectral broadening (line width), it shows up as inhomogeneous dephasing time in temporal signals such as photon echo, in which inhomogeneous broadening proportional to the *width* of the free induction decay component of a photon echo signal and its integrated function. This Section will provide calculations of one of the nonlinear optical techniques that eliminate inhomogeneous broadening, namely 2-pulse photon echo signal for model and real systems using eqs 23–28. Each photon echo signal will be followed by the corresponding linear homogeneous absorption spectrum to ratify its applicability and successful unmasking of the homogeneous vibrational structure.

The calculation presented in this Section will utilize the parameters that commonly arise in photosynthetic monomeric units, where the pigment–pigment interaction is too weak to be considered, as opposed to the exciton–phonon coupling strength, to demonstrate the accuracy and applicability of eqs 25–28. The subsequent calculations will involve photosynthetic dimeric complexes in which excitonic (pigment–pigment) coupling is finite and hence non-negligible as in *Rps*. *virides* bacterial RC. The forthcoming time-resolved 2-pulse photon echo signals will be computed using eq 28, whereas the integrated photon echo signals will be calculated using eq 27. However, the calculations will start with a time-resolved 2-pulse photon echo (PE) signal from a trial *model system for illustrative* purposes in which the following parameters are used in to calculate Figure 4: frequency mode *j* ω_*j*_ = 60 cm^–1^, *S*_*j*_ = 0.6, γ_*j*_ = 10 cm^–1^ (for the Lorentzian distribution of phonons), and σ_*j*_ = 3.5 cm^–1^ (for the Gaussian distribution of phonons) with a width for the ZPL Γ_ZPL_ = 3 cm^–1^ due to the electronic decay time of 1.77 ps at 1 K. Figure 4 and the forthcoming PE signal calculations will employ an inhomogeneous broadening Δ ∼ 107 cm^–1^. Furthermore, Figure 4 will be supplemented with the corresponding linear homogeneous absorption line shape in Figure 5 to ratify the structure of the time-resolved PE signal of the above systems,. Figure 4 shows that the PE signal starts with a strong free induction decay (as echo signals normally do) followed by quantum beats, each of which carries an asymmetric decay (made of a mixture of exponential and Gaussian decays, the width of each is stated above) and is centered around the period corresponding to ω_*j*_, tapering off with a long decaying tail due to the electronic decay (ZPL broadening in frequency domain). This vibronic structure in Figure 4 is composed of electronic decay (ZPL broadening) and quantum beats (asymmetric phonon profiles) appearing in the signal. The free induction decay in the inset of Figure 4 is cut off to better reveal the vibronic quantum beats and electronic decay. While τ signifies the time delay in femtoseconds (fs) between the two ultrashort pulses the foregoing and forthcoming figures, t stands for the observed time, which is also measured in fs. Figure 5 was calculated by taking the Fourier transform of eq 18 using the same parameters as in Figure 4 to show the homogeneous vibrational structure masked beneath the heterogeneous structure of amorphous proteins, thereby ratifying the vibronic structure revealed by the PE in Figure 4. Figure 5 starts with a sharp peak (ZPL) at 0 cm^–1^ followed by PSB composed of 1-phonon profile (0–1 transition) and 2-phonon profile (0–2 transition) appearing at 60 and 120 cm^–1^, respectively. As such, the first beat in Figure 4 corresponds to 1-phonon profile (0–1 transition) in Figure 5, whereas the second beat corresponds to 2-phonon profile (0–2 transition), and the long tail in Figure 4 signifies the ZPL in Figure 5.

**Figure 4:**
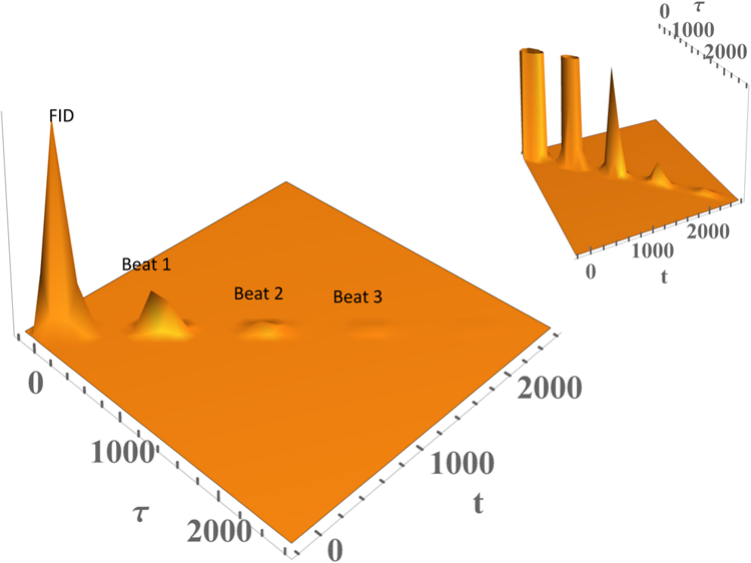
Time-resolved PE signal of a model system calculated with the following parameters: ω_*j*_ = 60 cm^–1^, *S*_*j*_ = 0.6, γ_*j*_ = 10 cm^–1^ (for half Lorentzian distribution of phonons), *T* = 1 K, Δ ∼ 107 cm^–1^, and σ_*j*_ = 3.5 cm^–1^ (for the half Gaussian distribution of phonons) with a width for the ZPL Γ_ZPL_ = 3 cm^–1^, caused by the electronic decay of 1.77 ps. The PE signal starts off with a sound free induction decay (FID) followed by quantum beats, each of which carries asymmetric decay (made of a mixture of exponential and Gaussian decays) and is centered at the period corresponding to 2π/ω_*j*_, tapering off with a long decaying tail, which reflects the electronic decay (ZPL broadening in frequency domain). The FID in the inset of Figure 4 is cut off to better reveal the vibrational quantum beats and electronic decay. While τ signifies the time delay in femtoseconds (fs) between the two ultrashort pulses, *t* stands for the observed time, which is also reported in fs. Beat 1, Beat 2, and Beat 3 represent the vibrational structure, and they would correspond progression members. These quantum beats become more pronounced as S increases. The electronic decay signifies electronic dephasing (ZPL width). This decay is long-lived in the low temperature limit.

**Figure 5:**
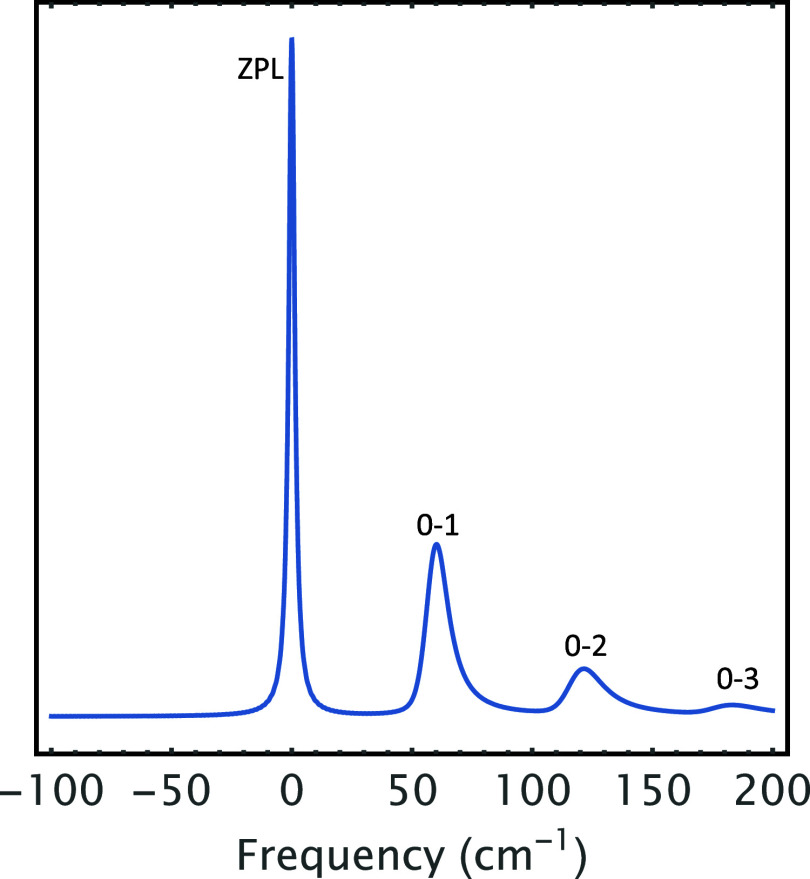
Linear homogeneous absorption line shape of the system in Figure 4, whereby the asymmetric distribution of phonons has been utilized to account for the asymmetric phonon profiles, which manifest themselves as quantum beats in Figure 4. Beat 1, Beat 2, and Beat 3 in Figure 4 correspond to (0–1), (0–2), and (0–3) transitions, respectively. Note that (0–1), (0–2), and (0–3) transitions, respectively, represent 1-, 2-, and 3-phonon profiles.

Figures 6 and 7 show calculations of the PE signal and the shape of homogeneous linear absorption lines, respectively, using parameters commonly found in monomeric pigment–protein complexes,^17^ whereby the asymmetric distribution of protein phonons is accounted for mode *j* ω_*j*_ = 30 cm^–1^, *S*_*j*_ = 0.5, γ_*j*_ = 20 cm^–1^ (for the Lorentzian distribution of phonons), and σ_*j*_ = 8.5 cm^–1^ (for the Gaussian distribution of phonons) with a width for the ZPL, Γ_ZPL_ = 3 cm^–1^. Figure 8 exhibits the integrated PE signal of Figure 6 to show the quantum beats better, whereas its inset is the integrated PE signal of Figure 6, both of which reflect a strong free induction decay, whose width is proportional to the measured inhomogeneous dephasing (often called inhomogeneous broadening in frequency domain) followed by a long electronic decay tail. The quantum beat in Figure 8 corresponds to the 1-phonon profile shown in Figure 7. Figures 9 and 10 utilize the parameters *Rps*. *virides* bacterial RC (special pair)^6–8,19^ to calculate the PE signal and the corresponding linear homogeneous absorption line shape, whereby the mean frequency mode (ω_*m*_ = 30 cm^–1^, *S*_*m*_ = 2.1, and γ_*m*_ = 55 cm^–1^) and the marker mode (ω_*sp*_ = 145 cm^–1^, *S*_*sp*_ = 1.0, and γ_*sp*_ = 50 cm^–1^) are included with the width of Γ_ZPL_ = 3 cm^–1^ due to electronic decay at T = 1 K. The parameters used to compute the absorption spectrum in Figure 10 are taken from the results published in ref [^6^]. The absorption spectrum in Figure 10 seems to be in a good agreement with that reported by Small et al.^6^ The inset of Figure 9 better shows the decay of free induction and quantum beats on a different scale, reflecting the vibronic structure of the bacterial RC. Figure 11 reveals the integrated PE signal of the photon echo in Figure 9 and its inset, whereby the intensity of the induction decay has been cut off for a better beat appearance. The prominent feature of the inset in Figure 11 is the close similarity between the quantum beats of the Bacterial RC and the phononic structure, both of which reflect the vibrational structure of the two modes present in the RC. This similarity was lucidly explored before^23^ in different systems. Finally, Figure 12 presents calculations of the *Rps*. *virides* bacterial RC PE signal at various temperatures using the excitonic coupling V_nm_ ∼ 300 cm^–1^. Evidently, as the temperature increases, the electronic decaying tail (ZPL) diminishes, and the quantum beats (vibrations) wash out in line with FCFs.

**Figure 6:**
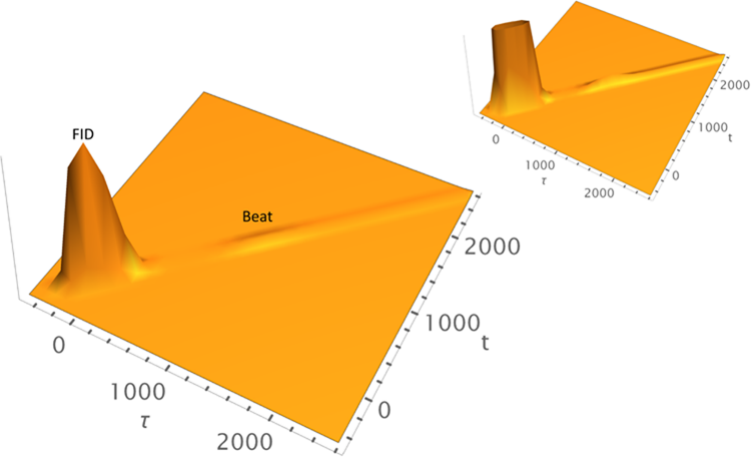
Time-resolved PE signal calculated using parameters commonly found in monomeric pigment–protein complexes, whereby the asymmetric distribution of protein phonons is accounted for mode *j* ω_*j*_ = 30 cm^–1^, *S*_*j*_ = 0.5, Δ ∼ 107 cm^–1^, γ_*j*_ = 20 cm^–1^ (for the Lorentzian distribution of phonons), and σ_*j*_ = 8.5 cm^–1^ (for the Gaussian distribution of phonons) with a width for the ZPL Γ_ZPL_ = 3 cm^–1^ at 1 K. The inset reveals the long electronic decaying tail in the time domain, which diminishes at high temperatures.

**Figure 7:**
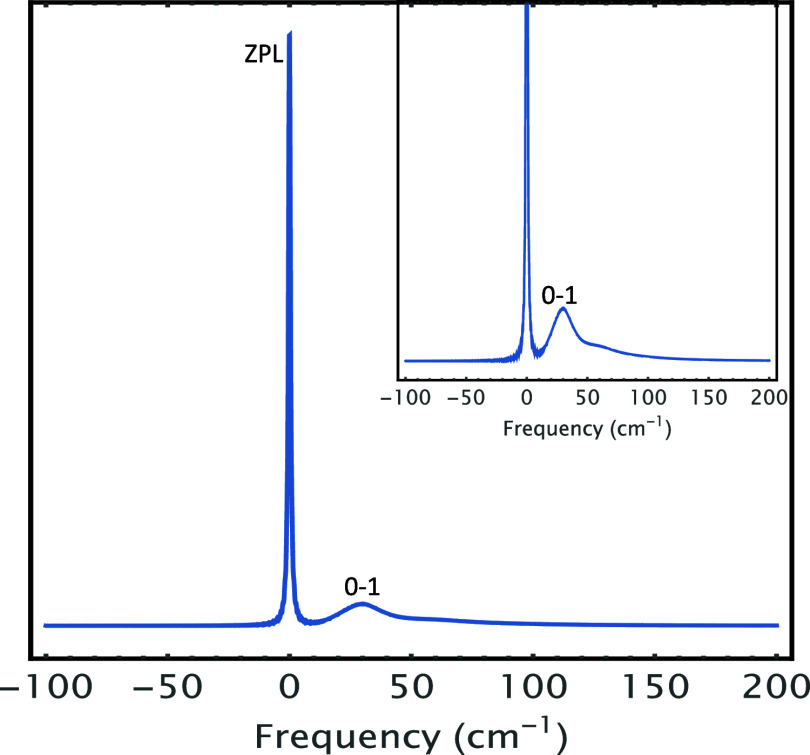
Corresponding linear homogeneous absorption spectrum of the monomer of the PE signal in Figure 6. The ZPL is the sharp peak here and, therefore, has been chopped off in the inset to show the asymmetric phonon sideband better. The quantum beat in Figure 6 corresponds to the 1-phonon profile here.

**Figure 8:**
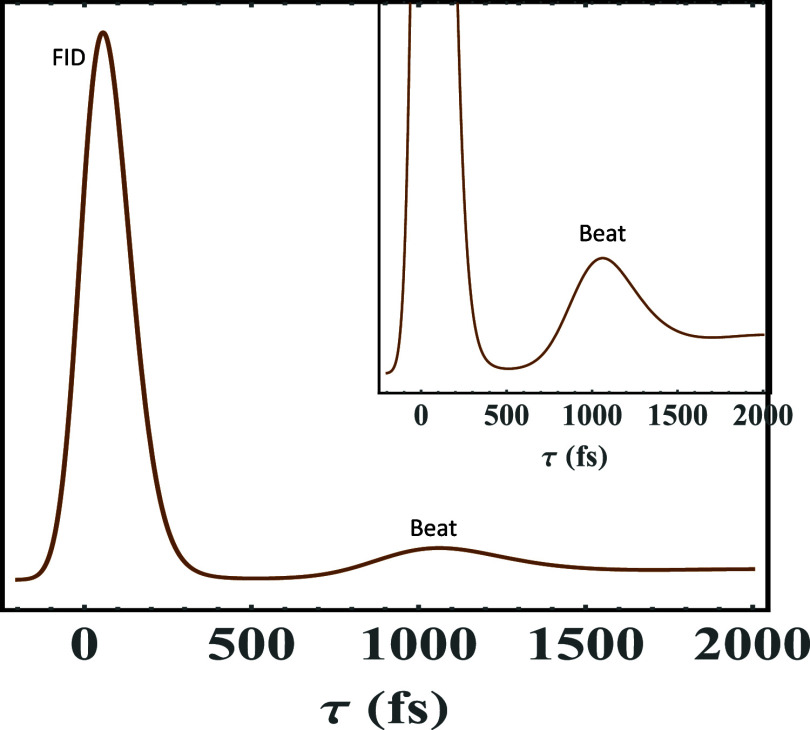
Integrated PE signal of Figure 6 better shows the quantum beats, whereas its inset is the integrated PE signal is that of Figure 6, both of which reflect a strong FID, whose width is proportional to the measured inhomogeneous dephasing (often called inhomogeneous broadening in frequency domain) followed by a long electronic decaying tail. The quantum beat in the inset corresponds to the 1-phonon profile in Figure 7.

**Figure 9:**
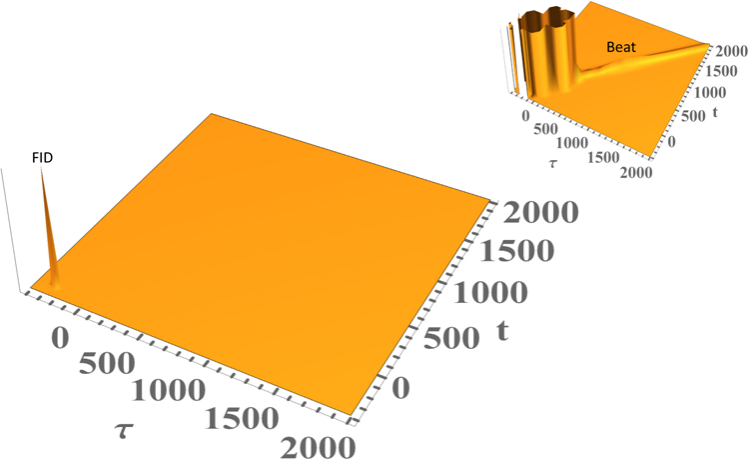
Time-resolved PE signal of *Rps. virides* bacterial RC special pair calculated using the following parameters: mean frequency mode (ω_*m*_ = 30 cm^–1^, *S*_*m*_ = 2.1, and γ_*m*_ = 55 cm^–1^) and the marker mode (ω_*sp*_ = 145 cm^–1^, *S*_*sp*_ = 1.0, and γ_*sp*_ = 50 cm^–1^) are included with the width of Γ_ZPL_ = 3 cm^–1^ due to electronic decay at *T* = 1 K and Δ ∼ 107 cm^–1^. The inset better displays the free induction decay and the quantum beats on a different scale, reflecting the vibronic structure of the bacterial RC.

**Figure 10:**
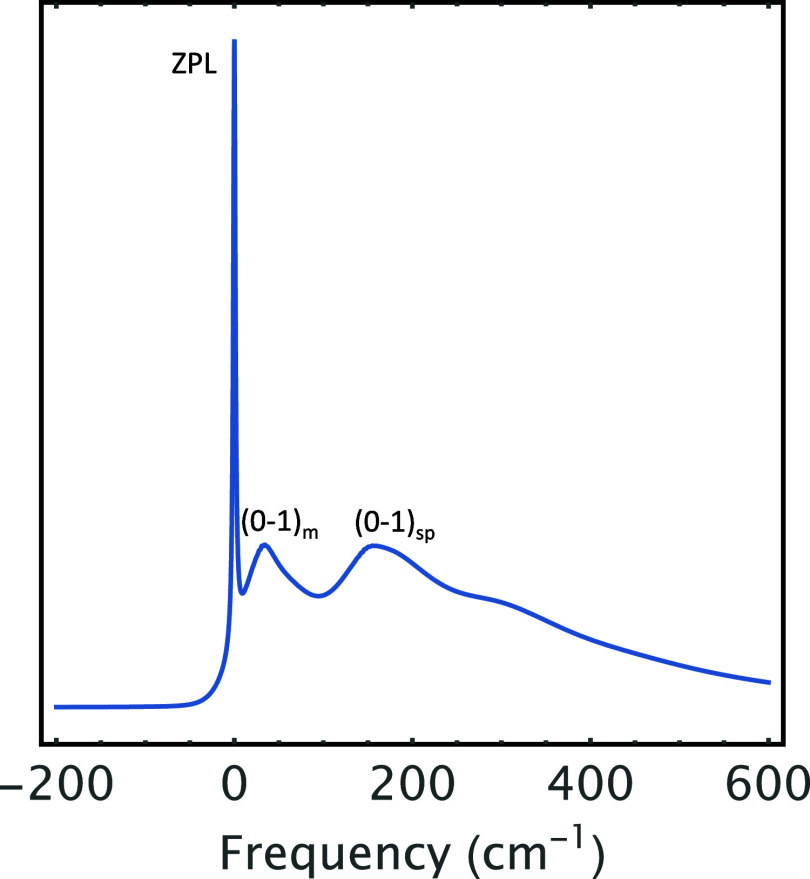
Corresponding linear homogeneous absorption line shape of *Rps. virides* bacterial RC special pair. The fundamental transition of each mode is labeled as (0–1)*_m_* for ω_*m*_ = 30 cm^–1^ and (0–1)*_sp_* for ω_*sp*_ = 145 cm^–1^, whereas the combination band is not well-resolved.

**Figure 11:**
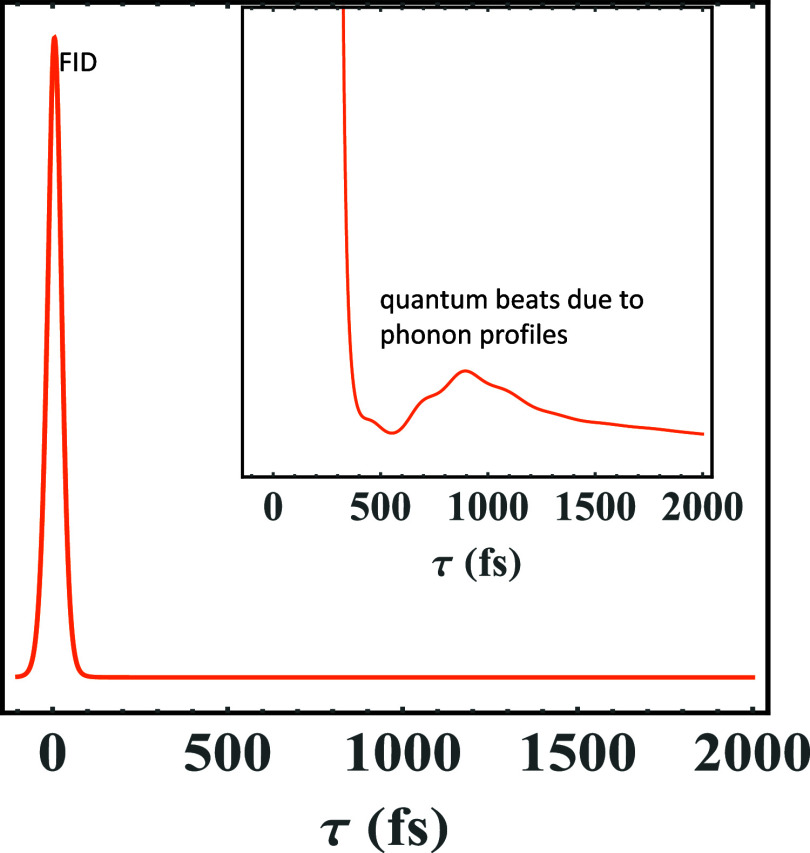
Integrated PE signal of the photon echo in Figure 9 along with its inset whereby the intensity of the FID has been chopped off for a better appearance of the quantum beats. The prominent feature of the inset is the close similarity between the quantum beats of the Bacterial RC and the phononic structure in Figure 10, both of which reflect the vibrational structure of the two modes present in the bacterial special pair.

**Figure 12:**
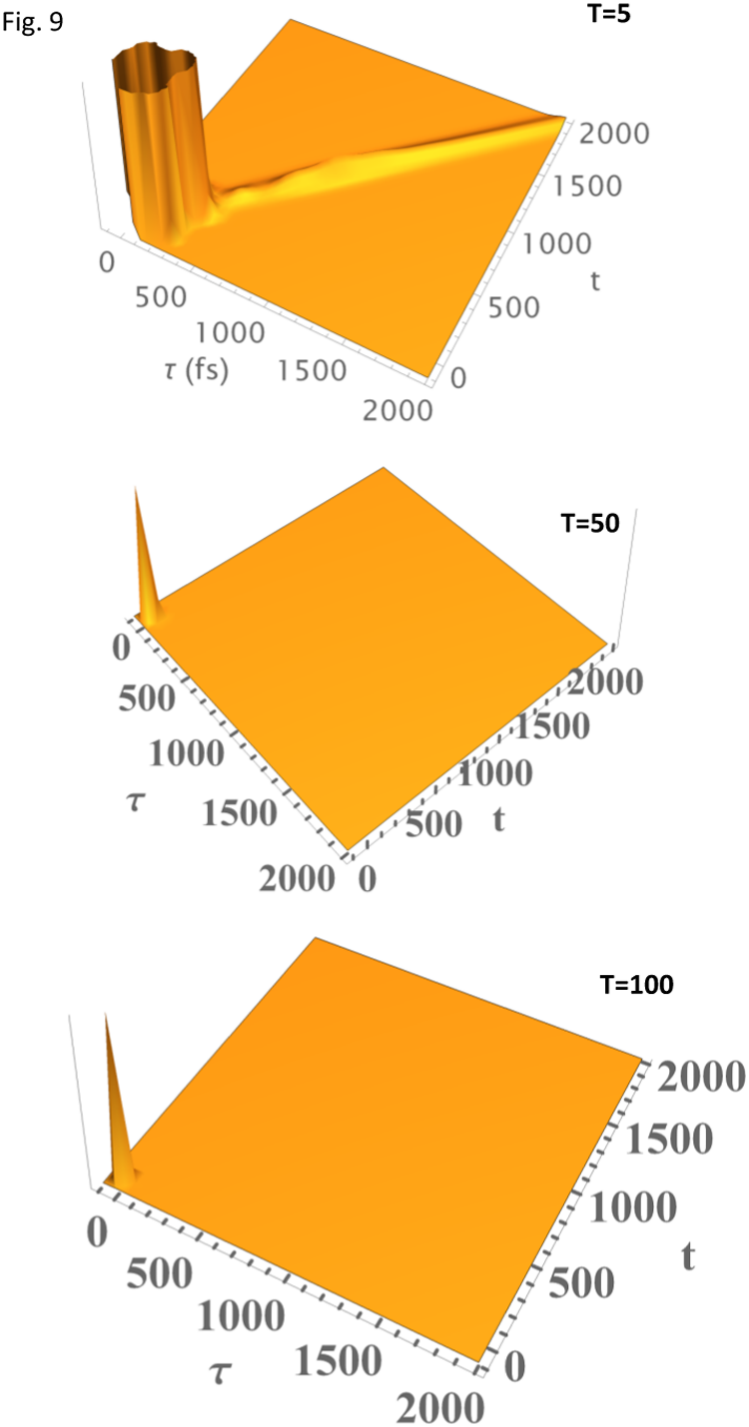
PE signal of *Rps. virides* bacterial RC PE signal at various temperatures using the excitonic coupling *J*_*nm*_ ∼ 300 cm^–1^. Evidently, as the temperature increases, the electronic decaying tail (ZPL broadening) diminishes, and the quantum beats (vibrations) are washed out.

## V Computational Methods

The equations in Sections 2 and III were coded in Mathematica to calculate the spectra and signals shown in the figures. Each relevant equation used is mentioned in the figure cation and the corresponding text.

## VI Concluding Remarks

This brief article has provided a derivation of the nonlinear electronic transition dipole moment-time correlation function in dimeric complexes, in which the phonon distribution is *asymmetric* for a multimode system, which distinguishes this work from other works. This work is expansive enough, starting from the time domain, to enable one to compute *all* nonlinear signals in both frequency- and time-domain while assuming *asymmetric* phonon distribution for multimode systems. The spectral density of the photosynthetic phonons leads to a phonon-sideband characterized by its *asymmetry*, caused by the unequal contribution from the photosynthetic phonons (bath) to the low- and high-energy sides of the optical signals. We have also shown that the spectral density manifests its asymmetry explicitly in the 1-phonon profile due to the intimate spectral connection between them, which will reflect in the entire phononic part of the absorption spectrum. This asymmetry is an essential contributing component to the exciton–phonon coupling strength and the phonon frequency distribution, thereby providing flexibility in modeling the degree of asymmetry/symmetry needed for the bath and imparting the capability of fine-tuning the nature of electron–phonon coupling caused by pigment–protein interaction.

Unlike other works,^17^ where only hole-burning and fluorescence line narrowing signals could be calculated for one mode, as including more modes would considerably slow down the computation. Furthermore, while those of HSF and Jankowiak et al.^17^ do not offer the capability to compute any of these nonlinear signals in the time domain, eqs 18 and 23 may readily be utilized to perform these calculations using nonlinear optical response theory, as shown in the text. As lucidly mentioned in the Introduction, our work eliminates the discrepancies and deficiencies posed by HSF and offers an alternative framework that exceeds the computational efficiency and spectroscopic probing capabilities of condensed systems. Numerical calculations have been reported to ratify the derivation in this document. These calculations have included linear homogeneous absorption spectra and 2-pulse photon echo signals with parameters of a model system, a monomer, and a dimer. Further, 2-pulse photon echo signals of the bacterial RC *Rps*. *virides* at different temperatures have also been reported to test the utility and applicability of this work further. The results are satisfactory, based on the assumptions made at the outset of this work.
